# Perioperative care bundle versus conventional perioperative care in pediatric patients undergoing minimally invasive digestive endoscopy

**DOI:** 10.12669/pjms.41.2.11369

**Published:** 2025-02

**Authors:** Liya Wang, Lijuan Zheng, Xiaobo Zhao, Xue Gong, Zhengyi Su, Shuokang Gong

**Affiliations:** 1 Liya Wang, Department of Endoscopy Room, Hebei Children’s Hospital, Shijiazhuang city, Hebei Province, 050000, China; 2 Lijuan Zheng, Department of Endoscopy Room, Hebei Children’s Hospital, Shijiazhuang city, Hebei Province, 050000, China; 3 Xiaobo Zhao, Department of Endoscopy Room, Hebei Children’s Hospital, Shijiazhuang city, Hebei Province, 050000, China; 4 Xue Gong, Department of Endoscopy Room, Hebei Children’s Hospital, Shijiazhuang city, Hebei Province, 050000, China; 5 Zhengyi Su, Department of Endoscopy Room, Hebei Children’s Hospital, Shijiazhuang city, Hebei Province, 050000, China; 6 Shuokang Gong, Department of Endoscopy Room, Hebei Children’s Hospital, Shijiazhuang city, Hebei Province, 050000, China

**Keywords:** Children, Minimally invasive digestive endoscopy, Pediatric patients, Perioperative care bundle

## Abstract

**Objective:**

To investigate the effect of perioperative care bundle in pediatric patients undergoing minimally invasive digestive endoscopy.

**Methods:**

This was a retrospective study using clinical records of pediatric patients who underwent minimally invasive digestive endoscopy surgery at Hebei Children’s Hospital from May 2020 to October 2023. Patients were divided into Care bundle group and Conventional care group based on the treatment received and matched for age, gender, and body mass index in 1:1 ratio. Perioperative vital signs, postoperative recovery, incidence of complications, and family members’ satisfaction with nursing care were analyzed.

**Results:**

A total of 98 pediatric patients were included, with 49 patients in each group. After surgery, diastolic blood pressure (DBP), systolic blood pressure (SBP), heart rate (HR), and mean arterial pressure (MAP) of both groups increased compared to preoperative values and were significantly lower in the Care bundle group compared to the Conventional care group (*P*<0.05). The time for anal exhaust, time for defecation, time to first out-of-bed activity, time to start eating after surgery, and length of hospital stay were shorter in the Care bundle than the Conventional care group (all *P*<0.05). The incidence of complications in the Care bundle group (4.08%) was lower than the Conventional care group (16.32%) (*P*<0.05). The overall family members’ satisfaction with the nursing care was considerably higher in the Care bundle group (93.88%) compared to the Conventional care group (81.63%) (*P*<0.05).

**Conclusions:**

Perioperative care bundle in pediatric patients undergoing minimally invasive digestive endoscopy was shown to be more efficacious than the conventional perioperative care.

## INTRODUCTION

Digestive endoscopy is the most reliable and commonly used diagnostic and treatment measure for gastrointestinal diseases in pediatric patients.^1^,^2^ Minimally invasive digestive endoscopy improves the health status of patients while limiting trauma associated with surgical intervention.^3^,^4^ Nevertheless, digestive endoscopy remains an invasive treatment measure.^5^ Additionally, pediatric patients have a thin gastrointestinal wall and a narrow lumen, which further increases the difficulty and risks of surgical treatment.^5^–^7^ Therefore, effective nursing interventions are crucial to ensure the quality and safety of minimally invasive digestive endoscopy in this group of patients.

Currently used conventional nursing measures mainly focus on nursing cooperation and routine health education, lacking systematic and targeted measures, which often results in a gap between the overall effect and clinical expectations.^8^,^9^ Care bundle, a concept developed by the Institute for Healthcare Improvement, refers to using a set of three to five evidence-based interventions to improve the quality of care for the population of focus.^10^ Such intervention is highly operable, simple, and clear, and can ensure the quality of nursing services and improve patient outcomes.^11^ Currently, care bundle has been demonstrated to be effective in adult patients undergoing appendectomy,^12^ adult appendectomy,^13^ and gastrointestinal bleeding,^14^ but research on children is limited, and even less in minimally invasive digestive endoscopy in children. In this context, the current study aimed to explore the intervention value of perioperative care bundle nursing in pediatric patients undergoing minimally invasive digestive endoscopy.

## METHODS

This was a retrospective study using clinical records of pediatric patients who underwent minimally invasive digestive endoscopy at Hebei Children’s Hospital from May 2020 to October 2023. Based on the treatment received, the patients were divided into Conventional care group (patients received routine nursing) and Care bundle group (patients received care bundle nursing on the basis of conventional nursing care.), and matched for age, gender, and body mass index (BMI) in 1:1 ratio.

### Ethical Approval:

All procedures conducted in this study involving human participants complied with the ethical standards of institutions and/or national research committees and the Helsinki Declaration (revised in 2013). Written informed consent was obtained from the patients or their legal guardians. This study was approved by the Hebei Children’s Hospital Medical Ethics Committee (No. HRL2023-221, Dated: October 23, 2023).

### Inclusion criteria:

- Patients with surgical indications receiving minimally invasive surgical treatment through digestive endoscopy.
- Guardians aware of the study and signed a consent form.
- The American Society of Anesthesiologists (ASA) classification of Grade I-II.
- Pediatric patients with the age range of 1-12 years old.

### Exclusion criteria:

- Patients with blood system diseases.
- Patients with organic lesions in important.
- Patients with immune system disease.
- Patients with congenital heart disease.
- Patients with digestive tract malformation.
- Patients/guardians with speech communication barriers.

### Conventional care group:

Patients in this group received routine nursing. Before the surgery, nursing staff provided detailed explanations of the minimally invasive surgical process and precautions for digestive endoscopy to the patient and their family members and assisted the child in preparing for fasting, water restriction, and other related preparations. During the operation, nursing stuff cooperated with the physician to perform relevant operations, routinely monitored vital signs, ensured unobstructed respiratory and fluid pathways, strengthened postoperative vital sign monitoring, and informed the family members of the patient of postoperative nursing precautions.

### Care bundle group:

Care bundle nursing was provided on the basis of conventional nursing care. An intervention team, including attending physician, deputy chief nurse, nurse supervisor, head nurse, nurse practitioner, and charge nurse was established. The team received systematic training, including preoperative education on minimally invasive digestive endoscopy, intraoperative nursing and cooperation skills, and postoperative observation. The intervention team members carefully reviewed the patient’s medical history and followed the principles of evidence-based medicine to establish a care bundle intervention protocol as follows.^10^

### Preoperative care:

Accompanied by the charge nurses, the team carried out health education activities for the patients and their families. Since the research participants were children, who are young and lack strong comprehension ability, nursing staff can comprehensively consider the personality characteristics and age of the children and select appropriate forms of communication, including pictures, toys, and multimedia videos. The team mainly introduced the approach and duration of minimally invasive digestive endoscopy, and the operating room environment. While deepening the patients and their families’ correct understanding of the surgical approach, the team can gain the trust of the patients and their families, establish a harmonious and good relationship, and thus obtain their understanding and cooperation.

### Intraoperative care:

- On the day of surgery, the nursing staff strictly checked the patient’s basic information and attached a wristband for identification. The patient was then accompanied by their family members to the operating room.
- After completing preoperative preparation, charge nurse was arranged to accompany the patient throughout the entire process to distract, alleviate psychological stress through physical touches, and promote a positive attitude. The team gave the child verbal praise or recognition through gestures (thumbs up) and physical contact (holding hands, lightly tapping the shoulders, and touching the head).
- Due to the thin walls of pediatric venous vessels, combined with preoperative fasting and water restriction, special care was taken when performing puncture operations to ensure a successful one-time puncture; two venous channels were left and properly fixed.
- Protective pads were placed at the contact area between the patient’s body and the operation table to protect the skin from damage and discomfort. Hot water bags and blankets were used to prevent hypothermia.

### Postoperative care:

- After surgery, the team ensured that the relevant patient information was correct and accurate, and the patient was returned to the ward. The handover work was completed at the bedside.
- The charge nurse who accompanied the child informed the family of the successful surgery and explained the purpose and necessity of indwelling the relevant catheter.
- The team frequently inquired about the patients’ feeling so that medication or physical analgesia could be given promptly in cases of severe pain. The team also encouraged the patients to get out of bed and move around early based on their physical condition in order to avoid complications such as urinary tract infections and lower limb thrombosis.

### Observation indicators:

- Perioperative vital signs, including diastolic blood pressure (DBP), systolic blood pressure (SBP), heart rate (HR), and mean arterial pressure (MAP).
- Postoperative recovery indexes such as time for anal exhaust, time for defecation, time to first out-of-bed activity, time to start eating after surgery, and length of hospital stay.
- Incidence of complications, including hypothermia, incision infection, incision bleeding, and abdominal pain.
- Nursing care satisfaction among the family members was measured based on a self-designed scale. The evaluation included nursing attitude, nursing timeliness, nursing skills, and hospital environment, with a total of 10 points: very satisfied (9-10 points), satisfied (6-8 points), and dissatisfied (<6 points). Satisfied and very satisfied scores were included in the calculation of the overall satisfaction.

### Statistical Analysis:

All data analyses were conducted using the SPSS software (version 26.0; IBM Corp, Armonk, NY, USA) and PRISM software (version 8.0; GraphPad, San Diego, USA). Quantitative data were represented as mean ± standard deviation (SD). An independent sample *t*-test was used for inter-group comparison, and a paired *t*-test was used for intragroup comparisons before and after surgery. Counting data were represent as number and percentage, and compared using Chi-square test or Fisher’s exact tests, as appropriate. *P*<0.05 indicated that the difference was statistically significant.

## RESULTS

**Fig.1:**
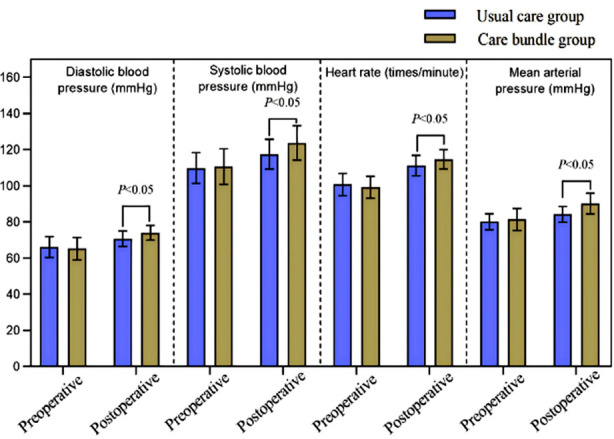
Comparison of perioperative vital signs between the two groups.

A total of 98 pediatric patients were included in this study, with 49 patients in each group. These patients included 54 male and 44 female, aged between two and 12 years (mean±SD, 5.90±2.38). There was no significant difference in baseline data between the two groups (*P*>0.05) (Table-I). Before surgery, DBP, SBP, HR, and MAP values were comparable in the two groups (*P*>0.05); After surgery, DBP, SBP, HR, and MAP levels in both groups increased compared to pretreatment values (*P*<0.05), and were considerably lower in the Care bundle group compared to the Conventional care group (*P*<0.05) (Fig.1). Care bundle nursing was associated with a significantly shorter time for anal exhaust, time for defecation, time to first out-of-bed activity, time to start eating after surgery, and length of hospital stay (*P*<0.05) (Table-II). Similarly, the incidence of complications was significantly lower in the Care bundle group (4.08%) compared to the Conventional care group (16.32%) (*P*<0.05) (Table-III). The overall rate of satisfaction with nursing care among the family members of the Care bundle group (93.88%) was higher than that of the Conventional care group (81.63%) (Table-IV).

**Table-I:** Comparison of General Information between Two Groups.

Item	Care bundle group (n=49)	Conventional care group (n=49)	t/χ^2^	P
***Gender***				
Male	25 (51.02)	29 (59.18)	0.368	0.544
Female	24 (48.98)	20 (40.82)		
Age (years)	5.71±2.17	6.00±2.49	-0.604	0.547
Body mass index (kg/m^2^)	15.54±1.29	15.2±1.22	1.354	0.179
***ASA grading***				
I	25 (51.02)	29 (59.18)	0.660	0.417
II	24 (48.98)	20 (40.82)		
***Surgical type***				
Colonoscopy surgery	27 (55.10)	32 (65.31)	1.065	0.302
Gastroscopy surgery	22 (44.90)	17 (34.69)		

**Table-II:** Comparison of postoperative recovery between two groups.

Group	n	Time for anal exhaust (hour)	Time for defecation (hour)	Time to first out-of-bed activity (hour)	Time to start eating after surgery (hour)	Length of hospital stay (day)
Care bundle group	49	22.86±3.27	36.04±4.69	39.16±3.04	22.63±4.85	5.65±1.28
Conventional care group	49	29.69±4.47	39.39±5.51	42.51±4.38	25.67±6.45	7.02±1.70
*t*		-8.641	-3.238	-4.390	-2.639	-4.490
*P*		<0.001	0.002	<0.001	0.010	<0.001

**Table-III:** Comparison of complications between the two groups.

Group	n	Hypotherm	Incision infection	Incision bleeding	Abdominal pain	Total incidence rate
Care bundle group	49	0(0.00)	0(0.00)	1(2.04)	0(0.00)	1(2.04)
Conventional care group	49	2(4.08)	1(2.04)	2(4.08)	3(6.12)	8(16.33)
*χ^2^*						5.995
*P*						0.036^b^

***Note:*** b represents Fisher’s exact test.

**Table-IV:** Comparison of nursing satisfaction in family members of the two groups.

Group	n	Very satisfied	Satisfied	Dissatisfied	The overall satisfaction
Care bundle group	49	33(67.35)	14(28.57)	2(4.08)	47(95.92)
Conventional care group	49	23(46.94)	17(34.69)	9(18.37)	40(81.63)
*χ^2^*					5.018
*P*					0.025

## DISCUSSION

This study showed that perioperative care bundle nursing care has a high application value in pediatric patients undergoing minimally invasive digestive endoscopy and is associated with improved perioperative vital signs, shorter postoperative recovery, and lower incidence of complications compared to conventional nursing care.

The study showed that care bundle nursing was able to effectively suppress blood pressure and HR fluctuations caused by invasive surgical procedures, maintain stable vital signs, shorten the time required for functional recovery, reduce the incidence of complications, and lower the hospitalization time of patients, which is consistent with previous studies. De Bijl-Marcus et al.^15^ explored the intervention value of care bundle nursing in 561 premature infants and confirmed that care bundle nursing can effectively reduce the incidence of cystic periventricular leukoplakia and stromal intraventricular hemorrhage, as well as the mortality rate. Osman et al.^16^ used care bundle nursing in pediatric patients in the intensive care unit and showed that it can reduce the incidence of ventilator-associated pneumonia. Together with our results, these observations suggest that the passive, not personalized nature of conventional care often fails to meet the physiological and psychological needs of surgical patients fully.

Moreover, potential risks may be difficult to detect in a timely manner with conventional care.^15^,^16^ As a result, targeted prevention and control measures cannot be taken, and timely adjustment of intervention measures cannot be made during clinical nursing, resulting in poor nursing outcomes. In contrast, care bundle nursing focuses on patient-centered interventions, integrates the personalized characteristics of patients, and provides targeted and systematic nursing services through evidence-based measures, which improves the quality of nursing services and prevents the occurrence of adverse events.^15^-^19^ Michel et al.^20^ applied care bundle nursing in pediatric intensive care units and confirmed that it could effectively reduce the risk of adverse events. These results further emphasized that the core concept of a care bundle is to comprehensively integrate a series of evidence-based nursing measures to improve the nursing service experience and treatment outcomes.^20^

Mohammad et al.^21^ found that compared to routine care, care bundle nursing can improve the health status of hypothermic newborns, ensuring their growth and development. Xu et al.^22^ also confirmed that bundled nursing can shorten the time required for clinical symptom relief in children, effectively improve their physical health status, and promote early recovery and discharge from the hospital. These findings are consistent with the conclusions of the present study. As pointed out by previous observations, young age and the pain associated with the disease negatively impact the psychological state of pediatric patients.^23^

In addition to the necessary nursing interventions, special attention should be paid to good interactive communication with the patient. Positive motivation and child-oriented guidance should be emphasized to bring patients closer and gain their trust and cooperation.^24^,^25^ While previous studies have confirmed the efficacy of care bundle nursing in clinical practice, there is still a lack of research on the application value of this nursing method in children undergoing minimally invasive digestive endoscopy.^15^,^16^,^20^,^21^ Our study bridges this gap by providing a practical reference and basis for clinical nursing in children undergoing this procedure.

The results of this study also showed that family members’ satisfaction with nursing was significantly higher in the Care bundle group. It is plausible that as care bundle nursing considerably shortens the recovery time of body function, reduces the occurrence of complications, and alleviates the impact of diseases on children and their families, the overall satisfaction with the treatment is higher.

Care bundles are relatively easy to develop and implement, and provide practitioners with a practical way to implement evidence-based practices to improve patient outcomes.^10^ In this study, we confirmed that the perioperative care bundles can improve patient outcomes in pediatric patients undergoing minimally invasive digestive endoscopy, which provides new ideas for quality control of clinical care for patients undergoing digestive endoscopy, provides children with the most optimized care services, and has important guiding significance for promoting the recovery of children.

### Limitations:

This is a single-center retrospective study with a small sample size. Additionally, the endoscopy was not performed by the same team, which may introduce variability and bias in the results of this study. Due to the limited communication and expression skills of the patients and their families, there may be a certain deviation in the satisfaction survey results. Higher-quality research is needed to validate the results of this study.

## CONCLUSION

Perioperative care bundle in children undergoing minimally invasive digestive endoscopy was shown to be more efficacious than conventional perioperative care as it is associated with more stable vital signs, shorter time to postoperative functional recovery, reduced risk of complications, and higher level of family satisfaction with nursing care.

### Authors’ Contribution:

**LW:** Conceptualization. Design and literature search.

**LZ and XZ:** Data collection, analysis, interpretation, critical review.

**XG:** Data analysis, interpretation, critical review.

**ZS:** Investigation, Critical review.

**LW and SG;** Writing, critical review & editing.

All authors have read the final manuscript and are responsible for integrity of the study.
